# Field Grand Challenge Frontiers in Antibiotics

**DOI:** 10.3389/frabi.2022.897071

**Published:** 2022-05-03

**Authors:** Stephen Henry Gillespie

**Affiliations:** Division of Infection and Global Health, University of St Andrews, St Andrews, United Kingdom

**Keywords:** antibiotics, drug development, clinical trials, genomics, diagnostics, epidemiology, antibiotic resistance

Paul Ehrlich has rightly been called the father of antibiotics. His seminal studies demonstrated three important principles about the use of antibiotics. The first was the concept of selective toxicity that underpins all our therapeutic choices. He also observed while he was developing the compound that became salvarsan, that continued exposure to the compound resulted in the emergence of resistance: the organism could no longer be killed. A complementary observation was that an agent with a different chemical structure and mode of action could kill the resistant organism (Ehrlich, 1909). Ehrlich received a Nobel Prize for this work upon which we all still stand (Fleming, 1929). In the subsequent years Salman Waxman also received a Nobel Prize for his discovery of streptomycin, Gerhard Domagk for his work on prontosil, and Fleming, Florrie and Chain for their pioneering work on the isolation of penicillin and enabling its production in large quantities. These and other notable awards indicate the critical contribution that antibiotics have made in the development of modern medicine. In the 1950s and 60s the discovery of the penicillin nucleus and the ability to chemically modify it opened a door to limitless possibilities to change the pharmacology and spectrum of activity and to transform treatment. This was an antibiotic golden age.

Discoveries continued apace, with the development of more active compounds with a wider spectrum of activity but, few new chemical classes were created. At the turn of the Millennium, it started to become apparent that a problem maybe growing, that of widespread antibiotic resistance. The specter of completely resistant organisms and a return to the pre-antibiotic age started to exercise the minds of not only physicians who had battled the problem in individual patients for many years, but of policymakers. A series of reports were made by national and international organizations of which the O'Neill report painted a dark future if we did not invest in preserving the antibiotics we have and devising new ones (O'Neill, 2016). The costs to health systems, it was predicted, could be $50bn per year. That there is a challenge in the sphere of antibiotics is universally acknowledged by international bodies, governments, and individual practitioners across a range of disciplines. Antibiotic resistance is rising globally at a time when the therapeutic pipeline is not providing new compounds. We urgently need to understand the problem and to identify solutions from a wide.

This brief summary of 100 years of antibiotic history lays out the agenda for those involved in antibiotic research today: understanding the epidemiology, the mechanisms of resistance and their transmission effective means of control, more rapid means of diagnosis and finding you antibiotics.

We have reason to be positive though as we have a promising toolset available to us with which to address the issue. Fred Sanger's invention of genome sequencing means that we now have unprecedented power to identify and track resistant organisms in the human population and we may also use that tool to understand the mechanisms of resistance (Sanger et al., 1977). The development of the polymerase chain reaction and similar techniques allow us to amplify microbial nucleic acids rapidly and make a diagnosis of identity and antibiotic resistance (Mullis, 1994). This is particularly valuable for organisms that are slow or difficult to grow such as mycobacteria (Telenti et al., 1993).

The rapid improvement in technology that has occurred means that we can investigate whole collections of organisms (Mutreja et al., 2011). By sequencing of large numbers of strains and their resistance genes, careful bioinformatic analysis allows us to understand multiple mechanisms of resistance, to track the transmission of resistant organisms and resistance genes across the globe (Török et al., 2013). Computer scientists are teaching us how to harness “big data” to address antibiotic development and resistance We can now start to understand the interconnectedness of pathogens, their interaction with human and intermediate hosts and fomites connections between human and animal populations demonstrating the importance of seeing antibiotics, their discovery, testing and implementation in a holistic setting: the one health environment (Ellabaan et al., 2021). We can now track organisms and the resistance determinant whether chromosomal or on mobile genetic elements with unprecedented clarity and in real time. This allows us to unravel complex transmission pathways and to understand the timeframe over which it occurs allowing plans and interventions to control transmission at local, national and international level (Parcell et al., 2017, 2020). Yet the scale of this challenge is enormous as we can conceive of each antibiotic-pathogen-human interaction of being a unique biological problem. As our new tools become available to answer old questions, we are increasingly recognizing that the new data come with more questions. Mathematics and computer science are contributing models that can test theories about resistance emergence and transmission (Ellabaan et al., 2021).

A whole panoply of diagnostic and epidemiological tools is needed to detect and track the evolution of resistant pathogens and, as has been recently demonstrated, the emergence of novel pathogens that affect every aspect of medicine and have a profound impact on antibiotic treatment and resistance (Santis et al., 2022). The global coronavirus pandemic has demonstrated the importance of effective defenses against infection. Not only was coronavirus a challenge but secondary pneumonias required additional antibiotic treatment. Thus, after the optimism of the 1980s when the assumption was that infectious diseases were a thing of the past, we now know through our genomic tracking that we are at risk of losing the use of antibiotics for many everyday infections. The threat of rising resistance has prompted many national and international reports. We are now starting to understand the human toll of resistance with an estimated 750,000 additional deaths due to resistant sepsis (Collaborators et al., 2022). Beyond that we see the economic toll on health services and support for those damaged by sepsis. Many pharmaceutical companies who developed and delivered novel antibiotics have left the market. Undoubtedly, this is a result of the success of antibiotics as patients take only a relatively short course and are, usually, fully cured needing no long-term prescription. This contrasts with, for example, anti-hypertensives where prescriptions are usually life-long. Given the up to 10 billion costs of bringing a new drug to market, these figures do not stack up financially. Alternative approaches to supporting antibiotic drug development must be found such as extending patent protection and regulatory waivers. Academia has an important role to play in innovation supported by institutions supporting spin-out or licensing deals that are realistic. The development of non-governmental organizations such as the TB Alliance and the Global Antibiotic Research and Development Partnership can drive and deliver new products (Spigelman and Gillespie, 2006; Piddock, 2021).

To rapidly plug the gap in the pipeline we may need to repurpose existing antibiotics and there is a growing research interest in using existing antibiotics in new combinations to address problematic organisms such as *Acinetobacter baumanii* (O'Donnell et al., 2021). There is, however, a pressing need to develop novel classes. An exciting area of innovation is to find antibiotic producing organisms from the marine environment. To achieve this, we will require new genomic tools to predict antibiotic producing organisms or genes likely to encode antibiotic production genes in organisms that are often difficult to grow or un-cultivatable. Genetic methodologies by selecting resistant mutants can help us understand the mechanism of action of novel compounds. Natural products from traditional medicines may prove to be another promising source of novel compounds harnessing modern techniques for compound separation (Bailey et al., 2019). These discoveries will need to be subject to structural analysis so that activity and spectrum can be optimized as it was once for the penicillins.

Developing new methodologies to speed the pre-clinical and clinical assessment of new antibiotics is a priority if we are to reduce the overall cost of the process and speed the time from discovery to implementation. Better *in vitro* and animal models that better mimic the pathology of infection can be used to select optimized compounds combinations and dosage that may progress to the clinical stage of testing (Bonnett and Davies, 2015; Bonnett et al., 2017; Kloprogge et al., 2019; Maitra et al., 2021). There is a growing interest in developing innovative clinical trials methodologies such as multi-arm multi-stage trials that enable multiple compounds to be assessed in early phase clinical trials simultaneously can be used to eliminate compounds with little change of success and to select compounds and regimens for the later more expensive large-scale phase three clinical studies (Boeree et al., 2017). With better mathematical modeling linked with innovative computer simulations it may be possible to model the natural history of infection in an individual end-organ or patient (Bowness et al., 2018). This would open the possibility of developing a virtual clinical trial that could test different combinations and dosages before patients are recruited. Some early examples of such approaches have already been published and this will be an important area of future research (Pitcher et al., 2020a,b).

We are increasingly recognizing the complexities of organizing effective antibiotic treatment. Uncontrolled access to antibiotics result in a rapidly increasing tide of resistance whereas in countries where access is tightly controlled resistance rates are lower. This emphasizes the global nature of antibiotic use and resistance: rising resistance in one place will inevitably produce resistance everywhere in our connected environment. Thus, we need to understand the detail of the drivers of antibiotic resistance. This includes, for example, health seeking behavior by individuals, the attitudes of health care providers about prescribing or dispensing antibiotics (Asiimwe et al., 2021). It is not merely a simple infection prescription problem, but the result of complex interactions between disease, patient, the environment, personal choices, wealth and opportunity among other factors that drive the prescription of antibiotics. It is, thus, by understanding the sociology of this clinical interaction that we will be able to develop effective antibiotic stewardship procedures that serve the needs of the individual patient and the larger community. How we organize, resource, and incentivise effective control is an open question that we must solve if we are to protect the new antibiotics that we hope to see developed.

To use the antibiotics that we have better we need to improve treatment outcomes to ensure prescriptions result in cure and that organisms are not able to develop resistance. This requires a detailed understanding of the way in which resistance mutations arise, survive and are transmitted overcoming the physiological barriers inherent in the process (Hjort et al., 2020; Roemhild et al., 2022). Such understanding would also support choices in the early stages of antibiotic development where compounds with a large physiological cost might be chosen against one with a lesser barrier. Better treatment will be aided by new methods to understand the pharmacokinetics of antibiotic to maximize the dose at the site of the infective lesion and novel testing methodologies are being adapted to monitor the effectiveness of antibiotic treatment in real-time. The development or enhanced pharmacological knowledge is of little benefit if we are unable to transmit the information to practitioners in a way that is appropriate for those clinicians with a heavy workload and this could, for example, include formularies supported by electronic tools and decision algorithms.

There is a paradox in practice: organisms divide very rapidly but our drug susceptibility testing results generally take at least 24 h to generate a result if they are not compromised by previous antibiotics. Better and more rapid drug susceptibility testing methodologies are an urgent priority. Recent reports indicate that it may be possible to obtain results in as little as thirty minutes which would have a transformative effect on clinical practice if the tools are available at affordable price and that the diagnostic system is adapted to the rapidity of the results (there is little point producing a very rapid result if the health care workers do not act on it) (Baltekin et al., 2017).

Although these developments give us unprecedented insight into resistance biology, such opportunities demand new epidemiological methodologies, analysis tools and clinical systems to optimize the impact on our research and to operationalise the methods in day-to-day practice. Understanding how antibiotics are used by physicians, other care givers, veterinarians, and the attitudes of patients and users is critical for using antibiotics effectively and controlling the emergence of resistance.

It is undeniable that the unrivaled legacy of early pioneers who developed antibiotics and that we have all benefited from is under threat. Yet there are significant reasons for optimism. We have started to recognize the problems and to research to understand how we can overcome it. This will require multi-disciplinary research across a spectrum of scientific, clinical, mathematical, sociological and veterinary and many other disciplines. New tools in genomics, mathematics computer science and chemistry among others mean that we well place to make progress and inaugurate a second antibiotic golden age.

## Author Contributions

The author confirms being the sole contributor of this work and has approved it for publication.

## Conflict of Interest

The author declares that the research was conducted in the absence of any commercial or financial relationships that could be construed as a potential conflict of interest.

## Publisher's Note

All claims expressed in this article are solely those of the authors and do not necessarily represent those of their affiliated organizations, or those of the publisher, the editors and the reviewers. Any product that may be evaluated in this article, or claim that may be made by its manufacturer, is not guaranteed or endorsed by the publisher.
